# Autologous macrophage therapy for liver cirrhosis: a phase 2 open-label randomized controlled trial

**DOI:** 10.1038/s41591-024-03406-8

**Published:** 2025-01-10

**Authors:** Paul N. Brennan, Mark MacMillan, Thomas Manship, Francesca Moroni, Alison Glover, Debbie Troland, Iain MacPherson, Catriona Graham, Rhona Aird, Scott I. K. Semple, David M. Morris, Alasdair R. Fraser, Chloe Pass, Neil W. A. McGowan, Marc L. Turner, Lynn Manson, Neil J. Lachlan, John F. Dillon, Alastair M. Kilpatrick, John D. M. Campbell, Jonathan A. Fallowfield, Stuart J. Forbes

**Affiliations:** 1 Division of Molecular and Clinical Medicine, University of Dundee, Dundee, UK; 2 Centre for Regenerative Medicine, Institute for Regeneration and Repair, University of Edinburgh, Edinburgh, UK; 3 Edinburgh Transplant Centre, Royal Infirmary of Edinburgh, Edinburgh, UK; 4 Aberdeen Royal Infirmary, Aberdeen, UK; 5 Scottish National Blood Transfusion Service (SNBTS), Edinburgh, UK; 6 Wellcome Trust Clinical Research Facility, University of Edinburgh, Edinburgh, UK; 7 Centre for Cardiovascular Science, University of Edinburgh, Edinburgh, UK; 8 Glasgow Royal Infirmary, Glasgow, UK; 9 Centre for Inflammation Research, Institute for Regeneration and Repair, University of Edinburgh, Edinburgh, UK

**Keywords:** Translational research, Liver cirrhosis

## Abstract

Cirrhosis is a major cause of morbidity and mortality; however, there are no approved therapies except orthotopic liver transplantation. Preclinical studies showed that bone-marrow-derived macrophage injections reduce inflammation, resolve fibrosis and stimulate liver regeneration. In a multicenter, open-label, parallel-group, phase 2 randomized controlled trial (ISRCTN10368050) in *n* = 51 adult patients with compensated cirrhosis and Model for End-Stage Liver Disease (MELD) score ≥10 and ≤17, we evaluated the efficacy of autologous monocyte-derived macrophage therapy (*n* = 27) compared to standard medical care (*n* = 24). The primary endpoint was the difference in baseline to day 90 change in MELD score (ΔMELD) between treatment and control groups (ΔΔMELD). Secondary endpoints included adverse clinical outcomes, non-invasive fibrosis biomarkers and health-related quality of life (HRQoL) at 90 d, 180 d and 360 d. The ΔΔMELD between day 0 and day 90 in the treatment group compared to controls was −0.87 (95% confidence interval: −1.79, 0.0; *P* = 0.06); therefore, the primary endpoint was not met. During 360-d follow-up, five of 24 participants in the control group developed a total of 10 severe adverse events, four of which were liver related, and three deaths (two liver related), whereas no liver-related severe adverse events or deaths occurred in the treatment group. Although no differences were observed in biomarkers or HRQoL, exploratory analysis showed anti-inflammatory serum cytokine profiles after macrophage infusion. This study reinforces the safety and potential efficacy of macrophage therapy in cirrhosis, supporting further investigation.

## Abstract (web summary)

Results from the phase 2 MATCH01 clinical trial of autologous monocyte-derived macrophage therapy for liver cirrhosis revealed no liver-related severe adverse events or deaths in the treatment group.

## Main

Liver disease causes two million deaths per year worldwide (representing one in every 25 deaths). Most deaths are related to complications of cirrhosis, including hepatocellular carcinoma (HCC)^1^. Globally, in 2017, there were 10.6 million prevalent cases of decompensated cirrhosis and 112 million prevalent cases of compensated cirrhosis^2^. Steatotic liver disease (SLD), which encompasses both metabolic dysfunction-associated steatotic liver disease (MASLD) and alcohol-related liver disease (ALD), and chronic viral hepatitis represent the dominant etiologies of chronic liver disease (CLD)^3^.

Cirrhosis is end-stage scarring (fibrosis) that can be caused by any form of chronic liver injury and is characterized by disruption of the normal liver architecture, organ dysfunction and high blood pressure in the portal vein and its branches (portal hypertension)^4^. Hepatic decompensation represents a clear inflection point in the trajectory of CLD, with median survival dropping from over 12 years in patients with compensated cirrhosis to approximately 2 years for patients with decompensated cirrhosis^5^. In addition to the risk of life-threatening complications, such as ascites, variceal hemorrhage and hepatic encephalopathy, cirrhosis is associated with a significant reduction in health-related quality of life (HRQoL) for patients and increased psycho-social burden on relatives and carers^6^.

There are no approved medicines for the treatment of cirrhosis^7^. Successful etiological treatment, such as antiviral therapy or sustained alcohol abstinence, can induce substantial fibrosis regression in patients with cirrhosis^8^ with potential improvement in portal hypertension^9,10^, liver function and even ‘recompensation’ of decompensated cirrhosis^11–13^. However, for patients with cirrhosis in whom cure or suppression of the primary disease is not possible, standard medical care is limited to the use of non-selective beta-blockers to mitigate risk of decompensation and death^14^, treatment of incident cirrhosis-related complications and liver transplantation if indicated. Due to the inexorable increase in the burden of end-stage liver disease (ESLD) and an ongoing shortage of donor organs for liver transplantation, alternative treatment strategies, such as cell therapies, are being explored.

Previous cell therapy approaches for patients with CLD, including hepatocyte transplantation and infusion of mesenchymal stem cells or hematopoietic lineages to stimulate liver regeneration and/or augment liver functional reserve, have demonstrated variable outcomes, particularly in patients with established cirrhosis^15,16^. However, preclinical studies have shown that bone-marrow-derived macrophage injections can reduce liver fibrosis while stimulating tissue regeneration and improving biochemical function^17,18^. We previously demonstrated the safety of peripheral infusion of ex vivo matured autologous monocyte-derived macrophages in patients with compensated cirrhosis in a phase 1 dose-escalation trial^19^. Additionally, although uncontrolled, secondary endpoint measurements suggested potential effects on liver function. Here we report the findings of a multicenter, phase 2 randomized controlled trial of autologous monocyte-derived macrophage therapy, compared to standard medical care, in adult patients with compensated cirrhosis.

## Results

### Baseline participant characteristics

**Fig. 1:**
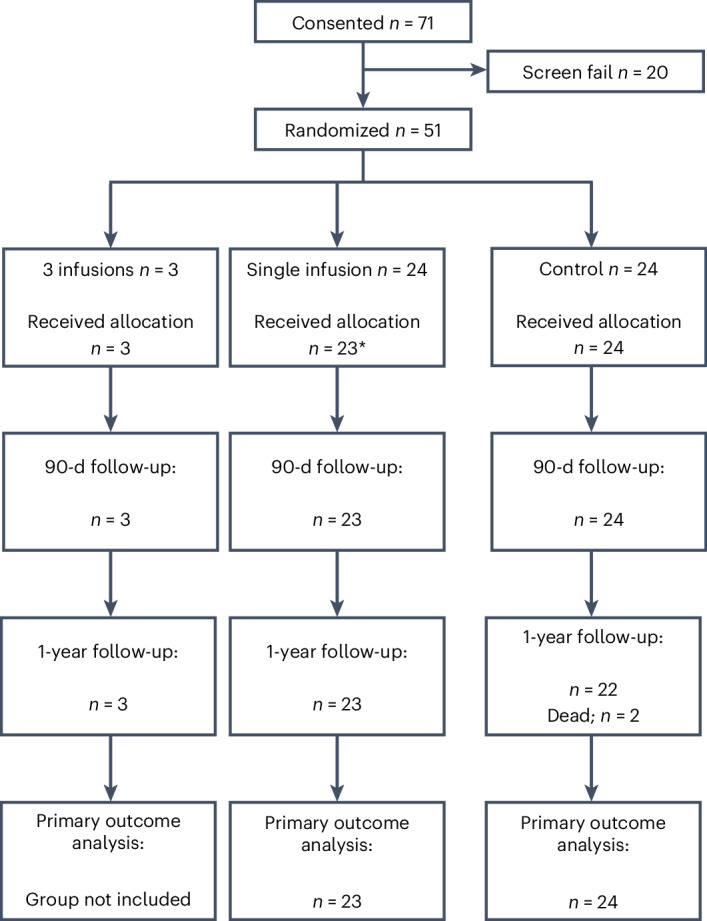
CONSORT diagram of the participant flow. The diagram is split by triple-infusion, single-infusion and control groups.

Seventy-one participants, recruited from three centers (Royal Infirmary of Edinburgh, Edinburgh, UK; Ninewells Hospital, Dundee, UK; Glasgow Royal Infirmary, Glasgow, UK), underwent screening as presented in the CONSORT diagram (Fig. 1) between 17 September 2017 and 26 November 2021, with the last patient completing follow-up on 24 August 2022. There were 20 screening failures, and 51 individuals were randomized, three of whom received the triple-infusion investigational medicinal product (IMP) regimen, with all subsequent participants receiving a single infusion. A single participant dropped out from the treatment arm due to vascular access problems before apheresis. Baseline participant characteristics are summarized in Table 1.

**Table 1:** Baseline demographics and clinical characteristics of trial participants

			Single infusion		Control		Total		*P*
			*N* (*n* = 24)	%	*N* (*n* = 24)	%	*N* (*n* = 48)	%	
Demographic data	Sex	Male	16	67	14	58	30	63	0.7661
	Age	Mean (s.d.)		57.8 (10.6)		60.7 (7.6)		59.3 (9.2)	0.2938
	Height (cm)	Mean (s.d.)		169.7 (9.5)		170.6 (9.7)		170.1 (9.5)	0.7395
	Weight (kg)	Mean (s.d.)		86.9 (19.7)		93.7 (19.4)		90.3 (19.6)	0.2395
	BMI	Mean (s.d.)		29.9 (5.3)		32.2 (6.8)		31.1 (6.1)	0.1976
	AFP (U l^−1^)	Median (Q1, Q3)		3.5 (2.5, 5.0)		3.0 (3.0, 5.0)		3.0 (3.0, 5.0)	0.8806
	VCTE (kPa)	Mean (s.d.)		33.0 (15.7) (*n* = 19)		40.6 (18.3) (*n* = 18)		36.7 (17.2) (*n* = 37)	0.1822
Laboratory data	Albumin (g l^−1^)	Mean (s.d.)		34.6 (3.8)		31.5 (5.2)		33.1 (4.7)	0.0246
	ALT (U l^−1^)	Mean (s.d.)		32.0 (16.0)		37.7 (27.3)		34.8 (22.3)	0.3862
	Bilirubin (μmol l^−1^)	Mean (s.d.)		33.0 (14.3)		36.1 (14.5)		34.6 (14.3)	0.4675
	INR	Mean (s.d.)		1.3 (0.1)		1.2 (0.2)		1.3 (0.2)	0.0938
	Creatinine (μmol l^−1^)	Mean (s.d.)		73.8 (19.4)		83.9 (25.1)		78.9 (22.8)	0.1282
	MELD	Mean (s.d.)		11.8 (1.8)		12.0 (1.7)		11.9 (1.7)	0.8053
	Platelets (10^9^ l^−^^1^)	Mean (s.d.)		96.2 (53.4)		115.3 (41.5) (*n* = 23)		105.5 (48.4) (*n* = 47)	0.1773
Liver disease	Dominant etiology	ALD	13	54	12	50	25	52	1.0000
		MASLD	7	29	8	33	15	31	1.0000
		PBC	1	4	3	13	4	8	0.6085
		Cryptogenic cirrhosis	1	4	1	4	2	4	1.0000
		Hemochromatosis	1	4		0	1	2	1.0000
		Hepatitis C virus	1	4		0	1	2	1.0000
	Other etiologies for this liver disease	Yes	7	29	1	4	8	17	0.0479
Past medical history	Liver	Ascites	10	42	10	42	20	42	1.0000
		Spontaneous bacterial peritonitis	1	4		0	1	2	1.0000
		Encephalopathy	7	29	8	33	15	31	1.0000
		Variceal bleeding	7	29	8	33	15	31	1.0000
		Other liver complications	1	4	6	25	7	15	0.0972
		Any previous hepatic decompensation	15	63	14	58	29	60	1.0000
	Cardiovascular	MI	1	4	1	4	2	4	1.0000
		Cerebrovascular accident		0	1	4	1	2	1.0000
		Other cardiovascular complications	2	8	4	17	6	13	0.6662
	Other	Asthma	4	17	2	8	6	13	0.6662
		COPD		0	2	8	2	4	0.4894
		Type 2 diabetes	9	38	10	42	19	40	1.0000
		IBD	1	4	4	17	5	10	0.3475
		Other rheumatologic conditions	1	4	1	4	2	4	1.0000
Alcohol use	Current use		8	33	9	38	17	35	1.0000
	Weekly intake	1–10 units	1	4	2	8	3	6	
		11–14 units	1	4	1	4	2	4	
		15–21 units		0	1	4	1	2	
		Less frequently	6	25	5	21	11	23	
	Abstinence duration	<6 months		0	1	4	1	2	
		1–12 months	4	17	4	17	8	17	
		1–2 years		0	2	8	2	4	
		>2 years	11	46	8	33	19	40	
		Missing	1	4		0	1	2	

Data include dominant etiology, comorbidities, recorded alcohol usage and periods of abstinence. Significance was computed using two-sided, two-sample *t*-tests or non-parametric equivalent, as appropriate. AFP, α-fetoprotein; BMI, body mass index; COPD, chronic obstructive pulmonary disease; IBD, inflammatory bowel disease; MI, myocardial infarction.

### Manufacture of cell therapy product

**Extended Data Fig. 1:**
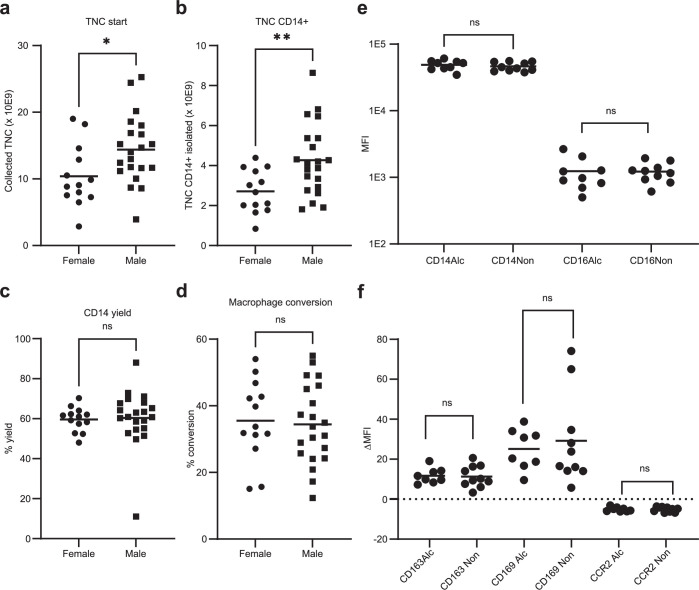
Isolation of monocytes and manufacturing of the MATCH01 product. **a-d**, Process robustness for manufacturing of the MATCH01 product. All products manufactured in the 23-participant single-dose group and the 3-participant triple-dose group. **a-b** Leukapheresis starting material (**a**, Total Nucleated Cell (TNC) collected from steady state leukapheresis from male and female participants * p = 0.0398; **b**, CD14+ monocyte content in steady state leukapheresis ** p = 0.0069). Two-sided unpaired *t*-test. **c-d**, Performance in manufacturing process (**c**, yield of CD14+ cells from the CliniMACS Prodigy selection procedure; **d**, final process yield of manufactured product for infusion, as a percentage of input CD14+ monocytes. ns = no significant difference by two-sided unpaired *t*-test. **e-f**, Phenotype of macrophages in final manufactured product for infusion, compared between alcohol and non-alcohol-related liver disease groups, 19 consecutive participants from single dose series. Two-sided unpaired *t*-tests. **e**, Expression of CD14 and CD16 in infused product between alcohol and non-alcohol-related liver disease groups. **f**, Change in relative expression of CD163, CD169 and CCR2 molecules on final product macrophages, compared to input monocytes. In all panels, bars represent group means.

**Extended Data Fig. 2:**
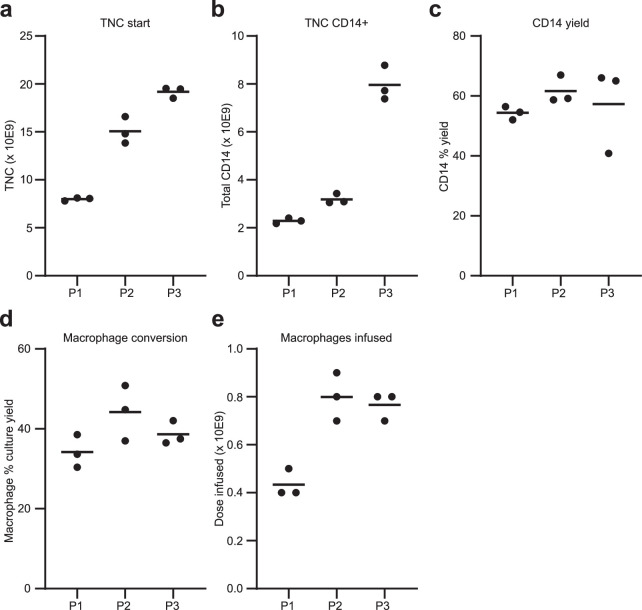
Intra-patient robustness of macrophage manufacturing process. Three participants (P1, P2, P3) donated leukapheresis three times at monthly intervals and received infusions of freshly manufactured macrophages. **a**, The total leukocyte content and the **b**, CD14 content of each donation remained constant between each donation timepoint. **c**, CD14+ selection efficiency and **d**, conversion to macrophages in the final product remained constant between each donation and comparable to the main group (Extended Data Fig. 1). **e**, Accordingly, the participants received infusions of comparable numbers of macrophages on each occasion, even though the product was manufactured in a unique production run per product.

Although the study population overall was relatively heterogeneous, the manufactured cell product generated from peripheral monocytes was homogeneous, irrespective of individual or etiological variance (Extended Data Figs. 1 and 2). Males donated significantly more leukocytes (*P* = 0.0398) and a corresponding number of CD14^+^ cells (*P* = 0.0069). CD14 selection performance was highly reproducible across all donations. There was a high degree of variability in the conversion efficiency of CD14^+^ to final product macrophages. We found highly homogeneous expression of CD14 and CD16 in infused product between alcohol-related and non-alcohol-related liver disease groups. Cell products also showed homogeneous upregulation of CD163 and CD169 scavenger molecules between disease etiologies. Relative expression of CCR2 is reduced in macrophages from the high levels seen in monocytes in both groups. The MAcrophage Therapy for liver CirrHosis (MATCH01) product is, therefore, highly comparable across trial donors with respect to surface protein expression.

### Primary endpoint

Here we present data for all patients who received cell treatment (one or three infusions) versus control patients. In extended data, we present the subanalysis for patients who received only a single infusion versus control.

**Fig. 2:**
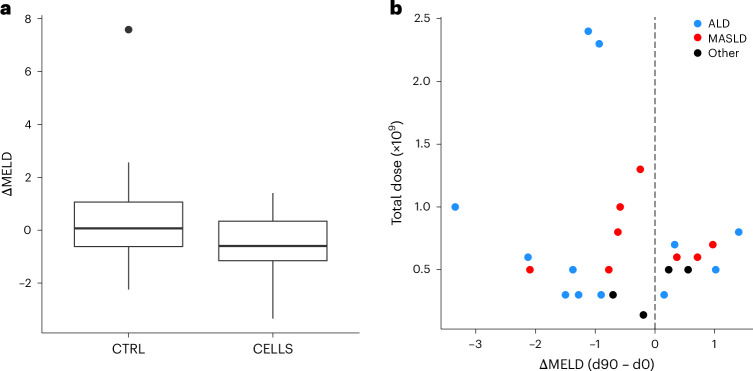
Baseline to day 90 ΔMELD score. **a**, Box plot of ΔMELD score, split by treatment allocation. Control (CTRL) group: *n* = 24 participants. CELLS group: *n* = 27 participants (includes both single-infusion and triple-infusion participants). Box plots are defined as first to third quartile (Q1, Q3), with center line representing the median. Whiskers extend to the lowest/highest values no further than 1.5× IQR from Q1/Q3, as appropriate. **b**, Plot of total cell dose (*n* × 10^9^) versus ΔMELD. Participants are color coded by dominant etiology (ALD, MASLD or Other). This plot includes all participants who received at least one infusion.

**Extended Data Fig. 3:**
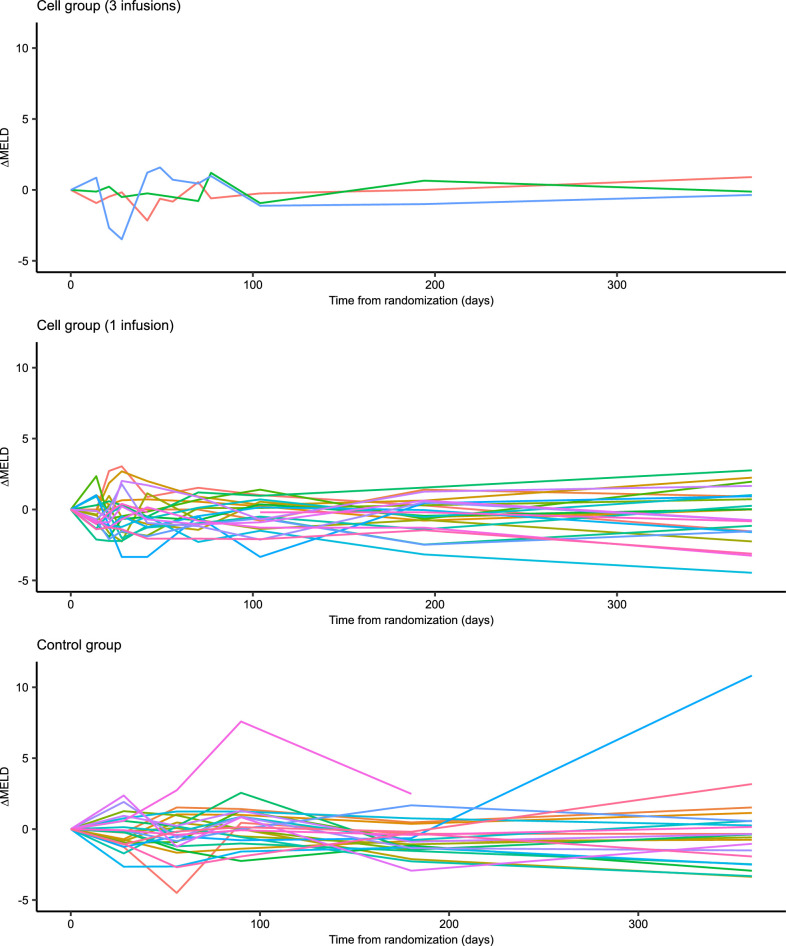
Plot of the baseline to day 360 changes in MELD score (∆MELD). Individual line plots are shown for each participant. Treatment allocation is separated into participants that received three cell infusions, single cell infusions and control (standard of care).

As described in the statistical analysis plan, the primary endpoint was the change in Model for End-Stage Liver Disease (ΔMELD) score assessed using a two-sample *t*-test (with Welch correction for unequal variance). The mean baseline MELD was 11.89 (s.d. ± 1.77) in the cell treatment group and 12.00 (s.d. ± 1.79) in the control group (*P* = 0.80). At day 90, MELD in the infusion treatment group was 11.43 (s.d. ± 2.00), and MELD in the control group was 12.40 (s.d. ± 3.15). This corresponds to a change in MELD of −0.48 (s.d. ± 1.10) versus +0.39 (s.d. ± 1.94) in the treatment and control arms, respectively. Overall, the ΔΔMELD between day 0 and day 90 in the treatment group compared to controls was −0.87 (95% confidence interval (CI): −1.79, 0.0; *P* = 0.06) (Fig. 2a). No clear cell dose–response or etiology-specific effect was observed (Fig. 2b). A summary of outcomes is presented for the participants who received macrophage infusion (one or three infusions) versus control (Table 2) or one macrophage infusion versus control (Extended Data Table 1). In Extended Data Fig. 3, we show the overall change in MELD for individual patients to day 360.

**Table 2:** Summary of primary and secondary outcome measures

	Single or triple infusion						Control						Difference in change			
	Baseline (*n* = 27)		90 d (*n* = 26)		Baseline to 90-d change (*n* = 26)		Baseline (*n* = 24)		90 d (*n* = 24)		Baseline to 90-d change (*n* = 24)					
	Mean	s.d.	Mean	s.d.	Mean	s.d.	Mean	s.d.	Mean	s.d.	Mean	s.d.	Mean	95% CI	*P*	Test
**MELD**	11.89	1.77	11.43	2.00	−0.48	1.10	12.00	1.79	12.40	3.15	0.39	1.94	−0.87	(−1.79, 0.04)	0.0601	Unequal
**UKELD**	50.97	2.05	50.61	2.34	−0.39	2.49	50.93	1.90	50.84	2.13	−0.09	1.97	−0.30	(−1.58, 0.98)	0.639	Equal
**Albumin**	34.19	3.97	33.96	3.67	−0.38	2.42	31.54	5.17	34.61	3.13	−0.17	2.33	0.62	(−0.71, 1.95)	0.33	Equal
**ALT**	33.59	16.13	33.00	17.63	−1.12	9.51	37.67	27.34	34.17	26.3	−3.5	7.58	2.38	(−2.53, 7.30)	0.33	Equal
**Bilirubin**	33.85	13.79	31.88	14.99	−2.15	8.69	36.08	14.47	35.13	16.08	−0.96	7.90	−1.20	(−5.93, 3.54)	0.6141	Equal
**INR**	1.30	0.15	1.26	0.15	−0.03	0.07	1.22	0.17	1.24	0.20	0.02	0.08			0.0861	Fisherʼs exact
**Creatinine**	72.59	18.59	72.58	20.59	0.19	6.94	83.88	25.10	92.46	47.38	8.58	34.12	8.39	(5.86, 10.92)	0.2255	Equal
**ELF**	11.55	1.07	11.72	0.90	0.27	0.88	12.33	0.99	12.24	1.43	−0.09	1.22	0.36	(−0.24, 0.96)	0.2328	Equal
**VCTE**	34.12	15.43	38.51 (*n* = 19)	18.84	5.97 (*n* = 19)	17.82	40.62 (*n* = 18)	18.29	31.46	15.18	−7.73	9.69	13.69	(3.97, 23.42)	0.0074	Unequal
**C3M**	15.60	4.89	15.37 (*n* = 25)	5.32	−0.53 (*n* = 25)	3.26	17.10 (*n* = 23)	7.92	17.11	7.66	0.15 (*n* = 23)	4.37	−0.68	(−2.90, 1.55)	0.5444	Equal
**Pro-C3**	29.53	14.83	24.78 (*n* = 25)	10.92	−3.82 (*n* = 25)	8.40	36.35 (*n* = 23)	20.47	33.23	21.67	−3.99 (*n* = 23)	7.51	0.17	(−4.48, 4.81)	0.9426	Equal

Data are included for all patients (single and triple infusions) versus controls. Significance was computed using two-sided, two-sample *t*-tests, controlling for equal variance as indicated. Significance for INR was computed using two-sided Fisher’s exact test. ALT, alanine aminotransferase.

In Supplementary Fig. 1, we show individual changes in MELD score from baseline to day 360 for patients stratified by dominant CLD etiology.

### Secondary endpoints

#### Safety and clinical outcomes

All participants within the control group completed the entire follow-up schedule. In those randomized to cell treatment, one patient was unable to undergo peripheral venous cannulation and was subsequently withdrawn from the study. There were no transfusion-associated reactions during or after administration of the macrophage infusions or in the 12-h post-infusion observation period. Overall, no dose–toxicity relationships were identified in the treatment group.

Within the 360-d follow-up period, a total of 291 adverse events (AEs) were recorded (Table 3a). In the control group (*n* = 24), a total of 104 AEs occurred in 23 participants, whereas, in the treatment group (*n* = 27, three triple infusions, 23 single infusions and one withdrawal), a total of 187 AEs occurred in 26 participants.

**Table 3:** AEs and SAEs within MATCH01 cohort

(a) Summary of treatment-emergent AEs and SAEs						
	Single infusion		Control		Total	
	(*n* = 24)	%^a^	(*n* = 24)	%^a^	(*n* = 48)	%^a^
Participants with AEs	23	96	23	96	46	96
Participants with AE defined as clinical event	5	21	5	21	10	21
Number of AEs	139		104		243	
Number of AEs defined as clinical event	5		8		13	
Participants with SAE	1	4	5	21	6	13
Participant with SAE defined as clinical event	0	0	3	13	3	6
Number of SAEs	1		10		11	
Number of SAEs defined as clinical event	0		3		3	

	Infusion (triple or single)		Control		Total	
	*N* (*n* = 27)	%^a^	*N* (*n* = 24)	%^a^	*N* (*n* = 51)	%^a^
Participants with AEs	26	96	23	96	49	96
Participants with AE defined as clinical event	5	19	5	21	10	20
Number of AEs	187		104		291	
Number of AEs defined as clinical event	5		8		13	
Participants with SAE	1	4	5	21	6	12
Participants with SAE defined as clinical event	0	0	3	13	3	6
Number of SAEs	1		10		11	
Number of SAEs defined as clinical event	0		3		3	

(b) Breakdown of SAEs within the MATCH01 clinical trial cohort		
	Treatment group (*n* = 27)	Control group (*n* = 24)
**Total SAEs (events)**	1	10 (five persons)
**Overview**	Collapse/alcohol intoxication	Ascites with AKI-HRS Encephalopathy Death
		Esophageal neoplasia
		dACLD HRS-AKI COVID-19 pneumonitis
		STEMI Femoral artery pseudoaneurysm Soft tissue infection
		Choledocholithiasis ERCP procedure
**Deaths**	0	3 (one patient with esophageal neoplasia was withdrawn on compassionate grounds before trial end) • Esophageal neoplasia • Decompensated liver disease • COVID-19 pneumonitis and decompensated liver disease

All AEs and SAEs, including ascribed clinical causality, as recorded in the MATCH01 trial cohort.

^a^Percentages are provided where appropriate. dACLD, decompensated advanced chronic liver disease; ERCP, endoscopic retrograde cholangiopancreatography; HRS-AKI, hepatorenal syndrome-acute kidney injury; STEMI, ST-elevation myocardial infarction. Note: no SAEs or clinical events were observed in the three participants who received three infusions.

Within the 360-d follow-up period, in the control group, five participants experienced a total of 10 severe adverse events (SAEs), four of which were liver-related SAEs. There were three deaths in the control group, two of which were related to decompensated liver disease and one of which was precipitated by coronavirus disease 2019 (COVID-19).

Within the 360-d follow-up period, in the treatment group (single or triple infusion), only one SAE occurred that was not considered as a clinical event. No liver-related SAEs or deaths occurred in the cell treatment group (Table 3b).

We compared the proportion of participants in the single-infusion group (proportion of individuals in the cell group with an AE = 0.95833) with one or more AEs against the proportion of participants in the control group (proportion of individuals in the control group with an AE = 0.95833). There was no evidence of a difference in the proportions (*P* = 1.000, Fisherʼs exact test due to small counts).

#### Enhanced liver fibrosis test

Assessment of changes in enhanced liver fibrosis (ELF) from baseline to day 90 in participants who received a treatment (triple and single infusion) showed a point estimate of 0.36 (95% CI: −0.24 to 0.9, *P* = 0.23) (Table 2), and participants who received a single macrophage infusion versus controls showed a point estimate of 0.3 (95% CI: −0.33 to 0.9, *P* = 0.34). No differences were observed in any of the individual ELF components (Extended Data Table 1).

#### PRO-C3 and C3M biomarkers

Using a two-sample *t*-test assuming equal variance in those who received treatment (triple or single infusion), no difference was observed from baseline to day 90 in either the type III collagen formation biomarker PRO-C3 (mean difference of 0.17 (−4.48 to 4.8), *P* = 0.94) or the type III collagen degradation biomarker C3M (mean difference of −0.68 (−2.90 to 1.5), *P* = 0.54) (Table 2). In participants who received a single macrophage infusion versus controls, no difference was observed from baseline to day 90 in either PRO-C3 (mean difference of 0.38 (−4.57 to 5.33) *P* = 0.87) or C3M (mean difference of −0.48 (−2.85 to 1.89) *P* = 0.68) (Extended Data Table 1).

#### United Kingdom Model for End-Stage Liver Disease score

The baseline to day 90 change in the United Kingdom Model for End-Stage Liver Disease (UKELD) score, comparing treatment (triple infusion or single infusion) with control groups using two-sample *t*-test showed no significant difference (−0.30 (95% CI: −1.58 to 0.9, *P* = 0.64)), and comparing single cell infusion to control groups using two-sample *t*-test also showed no significant difference (−0.36 (95% CI: −1.7 to 0.99, *P* = 0.59)).

#### Vibration-controlled transient elastography

Vibration-controlled transient elastography (VCTE) was undertaken longitudinally at specified study visits, and comparisons to baseline were made in patients with a complete set of measurements (28 patients in total; single (*n* = 15) and triple (*n* = 1) infusions versus control (*n* = 12)). Liver stiffness readings were technically valid (with interquartile range (IQR)/median <30% at all timepoints (day 0, day 90, day 180 and day 360)). At day 90, ΔVCTE showed an increase in liver stiffness in patients in the macrophage-treated group (mean +5.76 kPa, s.d. ± 19.17) and a decrease in those in the control group (mean −5.35 kPa, s.d. ± 9.03), although this difference was not statistically significant (*t*-test, *P* = 0.0536). Overall, liver stiffness decreased in both groups after day 90. Repeated ANOVA with timepoint and treatment as factors showed no interaction (*P* = 0.7976) but a significant effect of timepoint (*P* = 0.0090). Pairwise post hoc tests showed a significant difference in ΔVCTE at day 360 between macrophage-treated patients (mean +3.22 kPa, s.d. ± 19.72) and those in the control group (mean −12.71 kPa, s.d. ± 16.86) (*P* = 0.0268) (Supplementary Fig. 2).

#### Multiparametric magnetic resonance imaging

No significant difference was observed between baseline and day 90 multiparametric liver magnetic resonance imaging (MRI) measurements (Wilcoxon rank-sum exact test; corrected T1 (ΔcT1) *P* = 0.9170, ΔT2* *P* = 0.9012 and proton density fat fraction (ΔPDFF) *P* = 0.5978) (Supplementary Fig. 3 and Supplementary Tables 1–3).

#### Anti-inflammatory and pro-inflammatory cytokine data

**Fig. 3:**
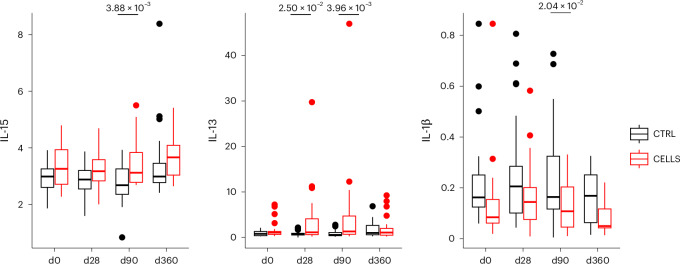
Longitudinal cytokine measurements. Box plots of anti-inflammatory and pro-inflammatory cytokines with significant effects due to macrophage treatment, split by timepoint and treatment. Control (CTRL) group: *n* = 23 participants (except d360 in all cytokines *n* = 21). CELLS group: *n* = 26 participants. Repeated ANOVA, two-sided, with pairwise two-sided post hoc tests. Box plots are defined as first to third quartile (Q1, Q3), with center line representing the median. Whiskers extend to the lowest/highest values no further than 1.5× IQR from Q1/Q3, as appropriate. d, day.

**Extended Data Fig. 4:**
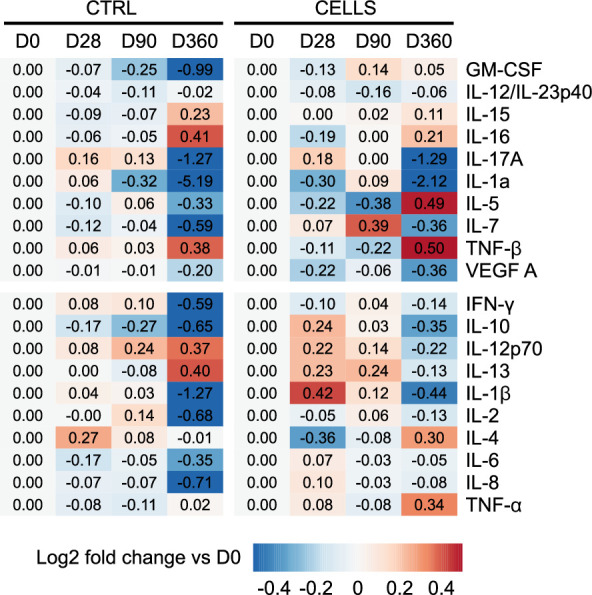
Summary of anti- and pro-inflammatory cytokine data. Heatmap of median log fold change for measurements of anti- and pro-inflammatory cytokines, compared to day 0, in treated and untreated groups.

Measurements of anti-inflammatory and pro-inflammatory cytokines were taken at specified study visits (day 0, day 28, day 90 and day 360). Repeated ANOVA with timepoint and treatment as factors showed no significant interaction for any cytokines. Cytokines showing a significant effect of treatment were subjected to subsequent post hoc pairwise testing. Interleukin (IL)-15 and IL-13 were significantly higher in macrophage-treated patients at day 90 (*P* = 3.88 × 10^−3^ and *P* = 3.96 × 10^−3^, respectively), and IL-1β was significantly lower in macrophage-treated patients at day 90 (*P* = 2.04 × 10^−2^) (Fig. 3). However, in all cases, the effect was not sustained beyond this timepoint. Median log fold changes compared to baseline are summarized in Extended Data Fig. 4; full cytokine panel data are provided in Supplementary Tables 1 and 2.

#### HRQoL assessment

Chronic liver disease questionnaire (CLDQ) scores were compared in participants with a complete set of measurements (day 0, day 90, day 180 and day 360). Cronbach’s α computed on domain means at each timepoint indicated high reliability of CLDQ scores (all participants ≥0.88; control participants ≥0.87; all macrophage-treated participants ≥0.89; single macrophage infusion participants ≥0.88). No difference was observed in the baseline to day 90 change for all macrophage-treated participants (*P* = 0.1610). Likewise, no difference was observed in the baseline to day 90 change in total CLDQ score for participants who received a single macrophage infusion compared to controls (Wilcoxon rank-sum test with continuity correction; *P* = 0.3354). Repeated ANOVA with timepoint and treatment as factors showed no significant interaction for any CLDQ domain. Pairwise post hoc tests showed a significant difference between treatment groups at baseline (day 0) in the abdominal symptoms domain (*P* = 3.09 × 10^−2^), with control patients scoring higher than macrophage-treated patients (mean 6.51 versus 5.64). However, we found no significant differences at subsequent timepoints (Supplementary Fig. 4 and Supplementary Table 3).

## Discussion

Hepatic decompensation heralds the development of multiorgan dysfunction, including portal hypertension, splanchnic vasodilation, left ventricular impairment and systemic immune dysfunction. Inflammatory mediators may underpin and potentiate nitric oxide–mediated capillary dysfunction, direct immunocytopathy and induce significant metabolic derangement with redistribution of essential nutrient precursors^20^.

For patients with cirrhosis in whom etiological therapy is unsuccessful or not possible, treatment options remain limited. Although numerous agents have been evaluated in clinical trials, there are no approved pharmacological therapies for reversing fibrosis or stimulating liver regeneration in the cirrhotic liver. Liver transplantation remains the only curative option for those with end-stage cirrhosis. Unfortunately, a substantial proportion of those referred for liver transplant assessment are ineligible, and, in the UK, approximately 12% die annually while on the waiting list^21^. Similarly, orthotopic liver transplantation carries substantial risk of morbidity and mortality, especially in relation to the need for lifelong immunosuppression.

Previous cell therapy studies used mesenchymal stem cells, hematopoietic stem cells and heterogenous cell populations, which included pro-inflammatory and pro-fibrotic cell lineages. Despite promising preclinical studies, randomized controlled trials of cell therapies in cirrhosis have, so far, been disappointing.

Macrophages have been shown to have a pivotal role in the regression of organ fibrosis^22–25^. Beyond their role in fibrosis, macrophages orchestrate signaling to the liver epithelial compartment and have pleiotropic effects on liver regeneration^17,26^.

This has led to their testing as a cell therapy in preclinical models of liver fibrosis^18,27–29^ and acute liver injury^30^. Monocyte-derived macrophages were developed as a Good Manufacturing Practice (GMP)-compatible cell therapy for human studies, and it was shown that monocytes from patients with cirrhosis could be differentiated ex vivo into macrophages with a similar phenotype to those from healthy volunteers^31,32^.

This prompted their testing in a phase 1 safety study of autologous macrophage therapy in cirrhosis, which demonstrated safety and, in this uncontrolled study, improvements in non-invasive tests of hepatic fibrosis in some participants after treatment, including transient elastography and serum biomarkers (ELF, PRO-C3 and C3M)^19^. These non-invasive tests were also examined in the present phase 2 randomized controlled trial. However, despite the promising phase 1 results, we did not observe significant improvements in these markers in the phase 2 study, although we note the relatively small sample size and challenge with use in cirrhosis, for which their utility is less clear^33,34^.

The MELD score, and particularly the change in MELD score (ΔMELD), predicts 3-month mortality in cirrhosis^35^. However, being a composite score, it is susceptible to fluctuations in any of its constituent elements. It was assumed that a ΔMELD of 1 point would represent a clinically meaningful change over the relatively short 90-d primary outcome interval. It is, however, recognized that liver remodeling is not a rapid or linear process, and substantial fibrosis regression would likely take far longer than 90 d to occur.

This trial did not achieve the primary MELD score endpoint. A difference was observed in the number of incident clinical outcomes between the treatment and control groups, despite exhibiting similar disease severity profiles at baseline. Although this trial lacked the statistical power to discern differences between the groups, there were more SAEs, including deaths, in the control group compared to those who underwent treatment.

In total, there were four incident liver-related events in two individuals in the control group. There was a further hospital admission for a hepatobiliary complication. Furthermore, in the control group, there were three deaths, two of which were associated with liver decompensation. In contrast, there were no liver-related SAEs or deaths in the treatment group. The only recorded SAE within the treatment group related to alcohol misuse in an individual who returned to harmful alcohol consumption and was admitted to hospital intoxicated and hypothermic. Although not statistically significant, it is an interesting observation, given the well-matched study arms and relative stability of the population at baseline.

This study can be contrasted with another phase 2 randomized controlled trial of cell therapy for liver cirrhosis (the REALISTIC study), with MELD scores in the range of 11–15.5 (ref. ^15^), that compared standard medical care (control) versus granulocyte colony-stimulating factor (G-CSF) alone versus G-CSF followed by leukapheresis and intravenous infusion of three doses of CD133^+^ hematopoietic stem cells. In this study, neither G-CSF nor G-CSF plus hemopoietic stem cell infusion improved liver dysfunction, and there was an increased frequency of AEs compared to standard medical care.

Designing clinical trials for cell therapies presents a unique set of challenges compared to traditional pharmaceutical trials, especially in patients with ESLD. Specific issues include product variability and standardization, the complexity of mechanisms of action, safety considerations and the regulatory landscape. Trial protocols for cell therapies are often complex, and recruitment and retention is difficult due to stringent entry criteria and significant dropout rates related to intensive long-term follow-up and the precarious nature of ESLD, which is associated with significant morbidity and mortality.

Notably, no concerning safety signals emerged in MATCH01, with no indications of macrophage activation syndrome or other pro-inflammatory sequelae in the short or medium term. Furthermore, no excess cases of cancer (including HCC) were recorded in the macrophage-treated group up to the day 360 timepoint. A long-term observational study extension will continue to monitor for clinical events for a further 36 months, of which an 18-month interim analysis has been presented^36^.

VCTE increased in the treatment group at day 56 after infusion of macrophages; however, this returned to baseline by the end of the follow-up period. We previously published on preclinical models of fibrotic liver disease in mice, whereby peripherally infused monocyte-derived macrophages migrate to, and localize within, the liver scar, where ongoing recruitment of circulating progenitor cells and liver-resident macrophages result in a transient pro-inflammatory phenotype, which may partly explain some of the transient increase in apparent liver stiffness measure (LSM)^18^. Notably, however, there is evidence of significant intra-individual and inter-operator variability that may explain some of the variance, particularly in patients with established cirrhosis, compared to less-established fibrotic disease. Therefore, we would caution against over-interpretation of VCTE within this context^37,38^.

We acknowledge several limitations inherent in this study. Regarding the trial design, it was considered unethical to undertake apheresis with reinfusion of a sham product to maintain participant blinding, necessitating an open-label design, which represents a limitation in this context. We also acknowledge the limitation that the open-label design necessitated accessors and staff being similarly unblinded. However, it is important to highlight that those participants in the control arm received extensive monitoring/follow-up according to the trial protocol, exceeding the level of care typically provided in real-world settings. The trial’s modest sample size is also a potential limitation, whereby significant outliers in either group could lead to overestimation or underestimation of true efficacy.

Regarding the etiology of liver disease between groups, allocation was by minimization with a random component to ensure a random allocation, and etiology was a factor in that minimization. The groups considered for etiology were ‘ALD’, ‘MASLD’ (the two major causes of cirrhosis in UK secondary care) and ‘Other’, and, based on this, we are balanced as far as possible. We recognize that there is a relative imbalance in the ‘Other’ etiology. However, there was not a significant imbalance in the number of participants with primary biliary cholangitis (PBC).

Additionally, the trial protocol lacked an objective assessment of alcohol intake (such as measurement of alcohol metabolites), relying on self-reported data from individual participants. This might result in underreporting of alcohol consumption, particularly in individuals with previously diagnosed ALD, which represented the leading etiology within the study cohort. Additionally, there is a confounding risk associated with alcohol consumption, as it might increase portal pressure even in cases where alcohol is not the primary etiology. One SAE was related to alcohol intoxication in a patient with known ALD, but underreported and covert alcohol use remains a challenging variable to objectively control for in clinical trials.

Although the MELD score was chosen based on previous studies, its suitability may be compromised in the context of a short follow-up interval. Although the study population mainly comprised patients with ALD and MASLD, which are the dominant CLD etiologies in Western countries, some participants had other liver conditions, including cholestatic diseases and viral hepatitis. Such heterogeneity might introduce an additional source of variation as distinct CLD etiologies exhibit different natural history profiles. Additionally, the potential effect of multiple (that is, coexistent) CLD etiologies was not accounted for, which could impact the risk of decompensation and mortality associated with synergistic pathologies. Despite the relative heterogeneity in CLD etiology, the manufacturing process yielded a uniform cell product, maintaining a consistent quantity of progenitor monocytes. Although specific cell concentration influences were not observed, variables such as volume of distribution and disease etiology probably contribute to variations in optimizing cell concentrations crucial for maximum efficacy, especially with multiple dosing schedules. The study population also exhibited limited geographical and ethnic diversity, predominantly comprising individuals of White Scottish descent.

Although the original trial protocol was designed to deliver three macrophage infusions, this was amended to a single-infusion regimen based on a pragmatic approach to recruitment and to limit the intensity of follow-up, which otherwise might have been excessively burdensome for participants. This change was approved by the trial steering committee and involved a substantial protocol amendment. Finally, as this study did not include pre-treatment and post-treatment liver biopsies, there was limited scope to investigate the mechanism of action of macrophage treatment. Nevertheless, we did observe significant (and durable) changes in circulating cytokine profiles between treatment and control participants. IL-15 levels were increased in macrophage-treated participants compared to controls. Notably, IL-15Rα signaling in hepatic stellate cells was shown to mediate a direct anti-fibrotic effect in mice^39^. In contrast, systemic levels of the potent pro-inflammatory cytokine IL1β were reduced after macrophage infusion. The IL-1 superfamily is a group of immunomodulatory cytokines with multiple pleiotropic effects. IL1β is a potent pro-inflammatory moiety involved directly and indirectly in potentiating fibrogenesis^40^ as part of activation of the inflammasome and represents a target for numerous liver pharmacotherapies. Serum IL-13 was increased in macrophage-treated patients versus controls; this anti-inflammatory cytokine also induces an ‘M2 like' phenotype in macrophages^41^.

Therefore, observed differences in all-cause (and, to a lesser extent, liver-related) outcomes after macrophage therapy are likely related to the modulation of immune dysfunction. Indeed, the importance of cirrhosis-associated immune dysfunction (CAID) is increasingly recognized and contributes to disease progression and multisystem consequences^42^.

In conclusion, this study reinforces the safety of autologous macrophage cell therapy in patients with compensated cirrhosis, suggests its therapeutic potential and supports further development of macrophage treatments in ESLD. Further trials are needed, particularly emphasizing the beneficial effects on clinical outcomes and elucidating the duration of response. Clearly, novel therapies are urgently needed for advanced CLD. This study builds upon growing understanding of the role of macrophages in liver repair and provides a potential new therapeutic approach for liver disease.

## Methods

### Regulatory approvals

The MATCH01 trial was approved by the Scotland A Research Ethics Committee (reference 15/SS/0121), the National Health Service (NHS) Lothian Research and Development Department and the United Kingdom Medicines and Healthcare products Regulatory Agency (MHRA). The trial was registered in the International Standard Randomized Controlled Trial registry (ISRCTN10368050) and the European Clinical Trial Database (EudraCT reference 2015-000963-15).

### Study design and participants

This was a multicenter, open-label, parallel-group, phase 2 randomized controlled trial to evaluate the efficacy of autologous monocyte-derived macrophage therapy, compared to standard medical care, in a population of male and female adults with compensated cirrhosis. The full study protocol was previously published as an open access article^43^.

Inclusion criteria included the following: age 18–75 years (inclusive) at time of screening; etiology—one or more of ALD, MASLD, PBC, cryptogenic cirrhosis, hemochromatosis, α1-antitrypsin deficiency and hepatitis C virus (if sustained viral response); diagnosis of cirrhosis—invasive or non-invasive criteria defined as one of previous histology confirming characteristic features of cirrhosis, transient elastography > 15 kPa and clinical and/or radiological features that, in the opinion of the investigator, correlate with a diagnosis of cirrhosis; and MELD score ≥10 and ≤17 at time of randomization. Exclusion criteria included the following: refusal or inability to give written informed consent; other causes of CLD not listed in the inclusion criteria (clinical judgment acceptable); portal hypertensive bleeding—active hemorrhage within 3 months requiring hospitalization (unless varices eradicated); presence of ascites —unless, in the opinion of the investigator, ascites is minimal (grade 1); hepatic encephalopathy—current or requiring hospitalization for treatment in the preceding 3 months; HCC or previous treatment of HCC; previous solid organ transplantation or use of immunosuppressive agents; any situation that, in the investigator’s opinion, may interfere with optimal study participation; presence of clinically relevant acute illness that may preclude on the basis of safety; presence or history of cancer, with the exception of adequately treated localized skin carcinoma, in situ cervical cancer or solid malignancy excised in total, with no recurrence (5-year interval); pregnancy or breast feeding; current enrollment in an interventional study; allergy to corticosteroids; and use of immunomodulatory therapy (including calcineurin inhibitors, thiopurines or methotrexate).

The initial trial protocol stipulated the administration of three infusions within the treatment arm. However, the requirement for repeated apheresis procedures imposed substantial demands on these participants. This presented a challenge to completing the trial within the scheduled timeframe. Consequently, after obtaining approval from the trial steering committee, the sponsor and the data monitoring committee, a revised single-infusion protocol was implemented. By the time of this modification, three participants had already received the triple-infusion regimen; the remaining 23 participants received a single infusion.

### Leukapheresis and cell manufacture

Participants underwent a standard leukapheresis protocol at the Scottish National Blood Transfusion Service (SNBTS) apheresis center in the Royal Infirmary of Edinburgh. Fractional blood material was then transported to the GMP facility at the Centre for Regenerative Medicine (Edinburgh, UK), and cell manufacture was performed as previously described^31^. These ex vivo matured autologous monocyte-derived macrophages exhibit a mature phenotype (CD14^+^/high 25F9 expression) plus the retention of high levels of markers associated with tissue repair and inflammation resolution (CD206, CD163 and CD169)^32^.

Participants randomized to the treatment arm received an infusion of the maximum yielded cellular concentration, up to a maximum of 1 × 10^9^ cells (day 0). If it was not possible to achieve 1 × 10^9^ macrophages per treatment, participants were reinfused with the quantity obtained, with the minimum cell concentration being 1.25 × 10^8^ cells as stipulated in the product release criteria designated by the MHRA.

### Randomization, minimization and blinding

This study employed a bespoke online randomization system developed by the Edinburgh Clinical Trials Unit to determine patient allocation. Participants were assigned to receive either standard medical care or a fresh dose of autologous macrophages at the maximum achievable dose in a 1:1 ratio based on a minimization algorithm using the key variable etiology of disease: ALD, MASLD or Other. To ensure that the allocation was random, participants were assigned to the group that minimized imbalance with a probability of 0.8. If a participant fell into two or more strata, then the dominant etiology (as determined by the treating physician) was used. Due to the nature of the intervention, neither participants nor staff could be blinded to the allocation of treatment. For some of the secondary endpoints, we maintained blinding of assessors, including those processing samples for serum biomarkers and analyzing imaging data.

### Study endpoints and assessments

#### Primary endpoint

The MELD score was originally devised to predict survival in patients with complications of portal hypertension undergoing elective placement of transjugular intrahepatic portosystemic shunt (TIPSS). The algorithm is based on serum creatinine, bilirubin and international normalized ratio (INR):

$$\begin{array}{l}{\rm{MELD}}({\rm{Original}},{\rm{Pre}}-2016)=\left(0.957\times \mathrm{ln}({\rm{Serum}}\; {\rm{creatinine}})\right.\\\left.+0.378\times \mathrm{ln}({\rm{Serum}}\; {\rm{bilirubin}})+1.120\times \mathrm{ln}({\rm{INR}})+0.643\right)\times 10.\end{array}$$

MELD scores were calculated at screening, randomization, cell infusion (day 0), safety visits (day 7 and day 14) and follow-up visits (day 28, day 56, day 90, day 180 and day 360). The relative change in MELD between those in the treatment group and the control group at baseline (day 0) and day 90 (ΔMELD 90 d) was the primary endpoint.

#### Clinical events

Regarding incident events occurring between study visits, patients were instructed to contact the Clinical Research Facility at any time if any new symptoms developed. In addition, at each study visit, the investigator asked participants about any AEs that may have occurred since the previous visit, details of which would then be recorded in the case report form and captured on the clinical database management system.

#### Serum fibrosis markers

The ELF test is a clinically validated immunoassay comprising the serum-derived biomarkers hyaluronic acid (HA), tissue inhibitor of metalloproteinase 1 (TIMP1) and amino-terminal propeptide of type III procollagen (PIIINP). The use of ELF and other non-invasive tests is recommended by the European Association for the Study of the Liver for risk stratification of patients with CLD^44^. ELF also has utility for prognostication, monitoring disease progression and treatment response. Indeed, a change in ELF score of 0.5 correlates with a single stage change in the Ishak fibrosis staging system^45^. ELF tests were performed at screening (if passed), day 28, day 56, day 90, day 180 and day 360 by iQur Ltd.

During extracellular matrix turnover, proteolytically cleaved matrix degradation fragments (or neoepitopes) are released into the systemic circulation. PRO-C3 and C3M are serum biomarkers that detect the formation and degradation of fibrillar type III collagen, respectively. Notably, PRO-C3 is an independent predictor of clinical outcomes in patients with advanced liver disease^46^. Fasting serum PRO-C3 and C3M levels were measured at screening (if passed), day 28 and day 90 by Nordic Bioscience.

#### UKELD score

The UKELD score, devised to predict the survival of patients listed for liver transplantation in the UK^47^, incorporates routine biochemical and hematological indices, including bilirubin, albumin, sodium and INR:

$$\begin{array}{l}{\rm{UKEL}}{\rm{D}}\; {\rm{Score}}=5.395\times {\rm{ln}}({\rm{INR}})+1.485\times {\rm{ln}}({\rm{creatinine}},{\rm{\mu }}{\rm{mol}}/{\rm{L}})\\+3.13\times {\rm{ln}}({\rm{bilirubin}},{\rm{\mu }}{\rm{mol}}/{\rm{L}})-81.565\times {\rm{ln}}({\rm{sodium}},{\rm{mmol}}/{\rm{L}})+435\end{array}$$

UKELD scores were calculated at screening, randomization, day 0, day 7, day 14, day 28, day 56, day 90, day 180 and day 360.

#### VCTE

FibroScan (Echosens) is a well-validated, non-invasive tool for liver fibrosis (VCTE), liver fat (controlled attenuation parameter (CAP)) and portal hypertension assessment. Furthermore, serial changes in liver stiffness by VCTE can predict the risk of clinical outcomes in patients with compensated advanced CLD^48^. FibroScan examinations were performed by trained operators at screening (if passed), day 90, day 180 and day 360, according to the manufacturer’s instructions.

#### Multiparametric MRI

LiverMultiScan (Perspectum) is a CE/CA-marked, FDA 510(k)-cleared, multiparametric liver MRI tool that quantifies fat (PDFF), iron content and fibroinflammatory activity (cT1). LiverMultiScan is sensitive to dynamic changes in disease activity and is prognostic of clinical outcomes^49^.

MRI scans were performed in a subset of *n* = 23 patients (cell recipients *n* = 10, controls *n* = 13) at screening (if passed) and day 90 according to the following protocol. After initial localization, a three-dimensional T1-weighted volumetric interpolated breath-hold examination (VIBE) with fat saturation was acquired to allow liver volume/segmentation. Sixty-four slices with an effective slice width of 3 mm were acquired. A single two-dimensional (2D) slice (6.0 mm) breath-held T2*-weighted multi-gradient echo was then selected through the center of the liver at isocenter to calculate a T2* map of the liver. A single 2D slice (9.0 mm) breath-held modified Look-Locker inversion recovery (MOLLI) was then acquired in the same anatomical position at isocenter to calculate T1 of the liver. Finally, five slices (10 mm with a slice spacing of 5 mm) of a 2D multi-gradient echo were acquired in the IDEAL configuration centered on the T1/T2* slice and were used to calculate the PDFF of the liver.

#### HRQoL

Impairment of HRQoL is described in most patients with advanced CLD. The CLDQ, which includes 29 items divided into six quality-of life-domains (fatigue, activity, emotional function, abdominal symptoms, systemic symptoms and worry)^50^, was performed at randomization, day 90, day 180 and day 360.

### Statistical analysis

#### Sample size

To detect a difference in the baseline to 90-d change in MELD score of 1 s.d. using a two-sided, two-sample test with a 5% level of significance, a sample size of 23 per group to detect the same level of difference with 90% power was required.

#### Primary endpoint

The baseline to 90-d change in MELD score was compared in the two treatment arms using a two-sided two-sample *t*-test. MELD scores calculated for each participant throughout the trial were used to calculate an area under the curve (AUC), and this was compared across the groups using a two-sample *t*-test.

#### Secondary endpoints

The baseline to 90-d changes in secondary endpoint measures were compared between treatment arms using a two-sided two-sample *t*-test or non-parametric equivalent as appropriate. Changes over the 1-year study period were used to calculate an AUC for each participant and compared across the groups using a two-sample *t*-test or non-parametric equivalent as appropriate. Repeated ANOVA with time and treatment as factors was used to compare VCTE data, CLDQ scores and cytokine measurements, followed by pairwise post hoc testing as appropriate.

#### Safety assessments

The number of participants experiencing SAEs and AEs in each of the two treatment arms was expressed as proportions. The difference in proportions was compared using a binomial test, and data were presented along with the 95% CI.

All analyses were carried out on an intention-to-treat basis.

Unless stated, numerical data are expressed as mean ± s.d. *P* values of less than 0.05 were considered statistically significant. Welch’s correction was applied for groups with unequal variances. We used SAS software version 9.4 (SAS Institute) and R version 4.2.3 (R Core Team) for all statistical analyses.

We primarily present analysis for all cell infusions (that is, patients receiving one or three infusions) versus control and, for completeness, include analysis of single infusion versus control.

### Trial oversight

The MATCH01 trial was an investigator-led study, funded by the Medical Research Council (reference MR/M007588/1) and sponsored by the Academic and Clinical Central Office for Research and Development (ACCORD) for NHS Lothian/University of Edinburgh. All study-related documents were designed by the trial team with input from ACCORD, an independent statistician (C.G.) and the SNBTS team. All participants enrolled in the study gave written informed consent, and the trial was conducted in accordance with the ethical principles of the Declaration of Helsinki and the International Council for Harmonization Guidelines for Good Clinical Practice. Trial oversight was provided by a trial steering committee and an independent data monitoring committee.

### Reporting summary

Further information on research design is available in the Nature Portfolio Reporting Summary linked to this article.

## Online content

Any methods, additional references, Nature Portfolio reporting summaries, source data, extended data, supplementary information, acknowledgements, peer review information; details of author contributions and competing interests; and statements of data and code availability are available at 10.1038/s41591-024-03406-8.

## Supplementary information

Supplementary Information Supplementary Figs. 1–4 and Supplementary Tables 1–3.

Reporting Summary

## Figures and Tables

**Extended Data Table 1:**
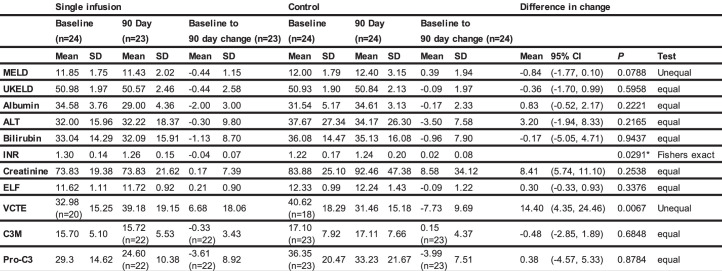
Summary of primary and secondary outcome data

Overview of the primary and secondary outcomes for single-infusion patients versus controls. Values shown are baseline, day 90 and baseline to day 90 change for each outcome measure in both single-infusion and control groups. Significance was computed using two-sided, two-sample *t*-tests, controlling for equal variance as indicated. Significance for INR was computed using two-sided Fisher’s exact test. MELD, model for end-stage liver disease; UKELD, United Kingdom model for end-stage liver disease; ALT, alanine aminotransferase; INR, international normalized ratio; ELF, enhanced liver fibrosis test; VCTE, vibration controlled transient elastography.

## Data Availability

Data in the main manuscript (and extended and Supplementary Information files) are presented where possible in aggregated form. Any data presented to illustrate individual patient performance have been de-identified and include only analysis of performance within the trial (such as MELD score). The datasets generated and/or analyzed during the current study are available from the corresponding author (S.J.F.) upon reasonable request, although restrictions may apply due to patient privacy and the General Data Protection Regulation.
