# Supplementary material for: Autologous macrophage therapy for liver cirrhosis: a phase 2 open-label randomized controlled trial
Source: Nat Med. 2025 Jan 10;31(3):979–87. doi: 10.1038/s41591-024-03406-8 (PMC11922741; doi:10.1038/s41591-024-03406-8)
Supplement: Supplementary file 1 — Supplementary Figs. 1–4 and Supplementary Tables 1–3. [file 41591_2024_3406_MOESM1_ESM.pdf]

nature medicine

Supplementary information https://doi.org/10.1038/s41591-024-03406-8

Autologous macrophage therapy for liver cirrhosis: a phase 2 open-label randomized controlled trial

In the format provided by the authors and unedited

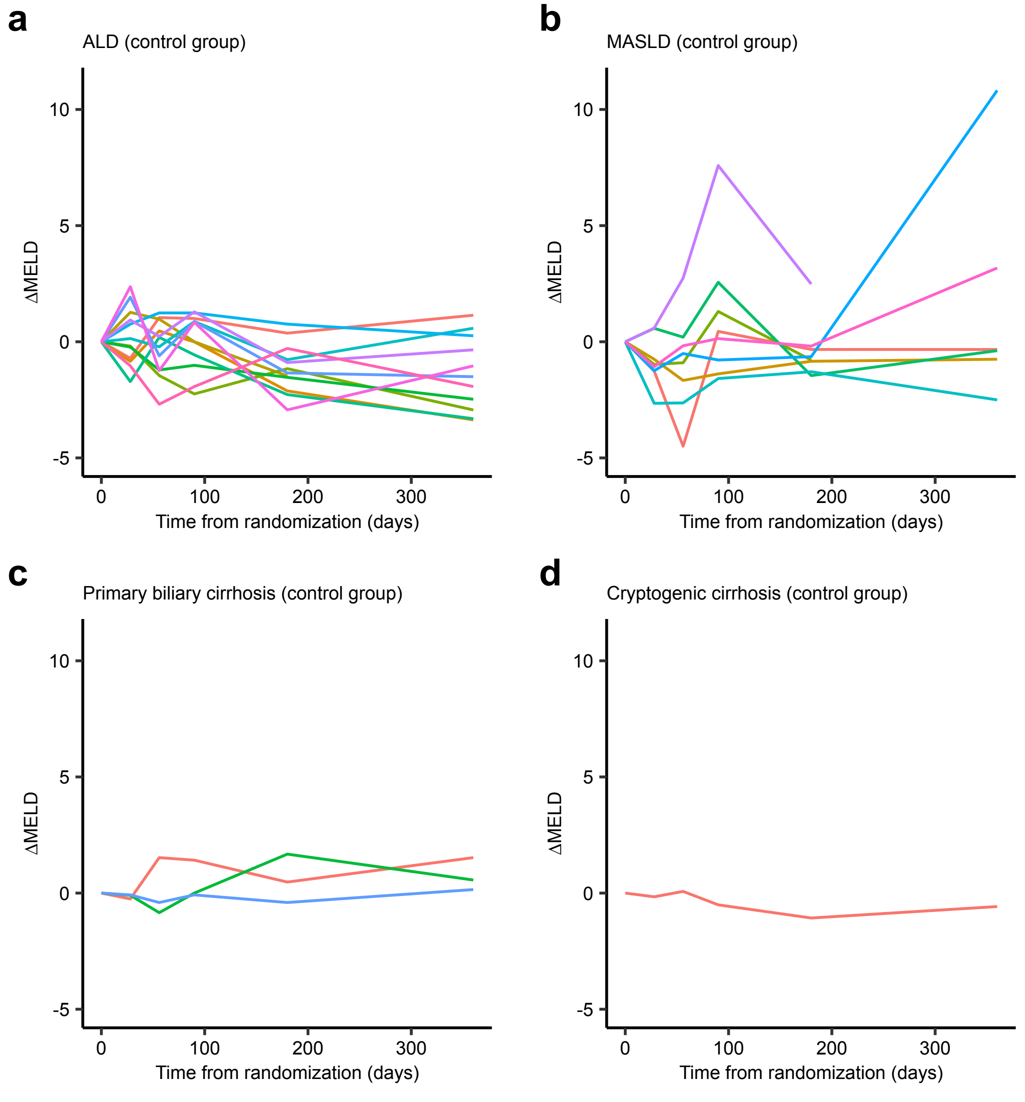

a
ALD (control group)
b
MASLD (control group)
c
Primary biliary cirrhosis (control group)
d
Cryptogenic cirrhosis (control group)
ΔMELD
Time from randomization (days)
10
5
0
-5
0
100
200
300

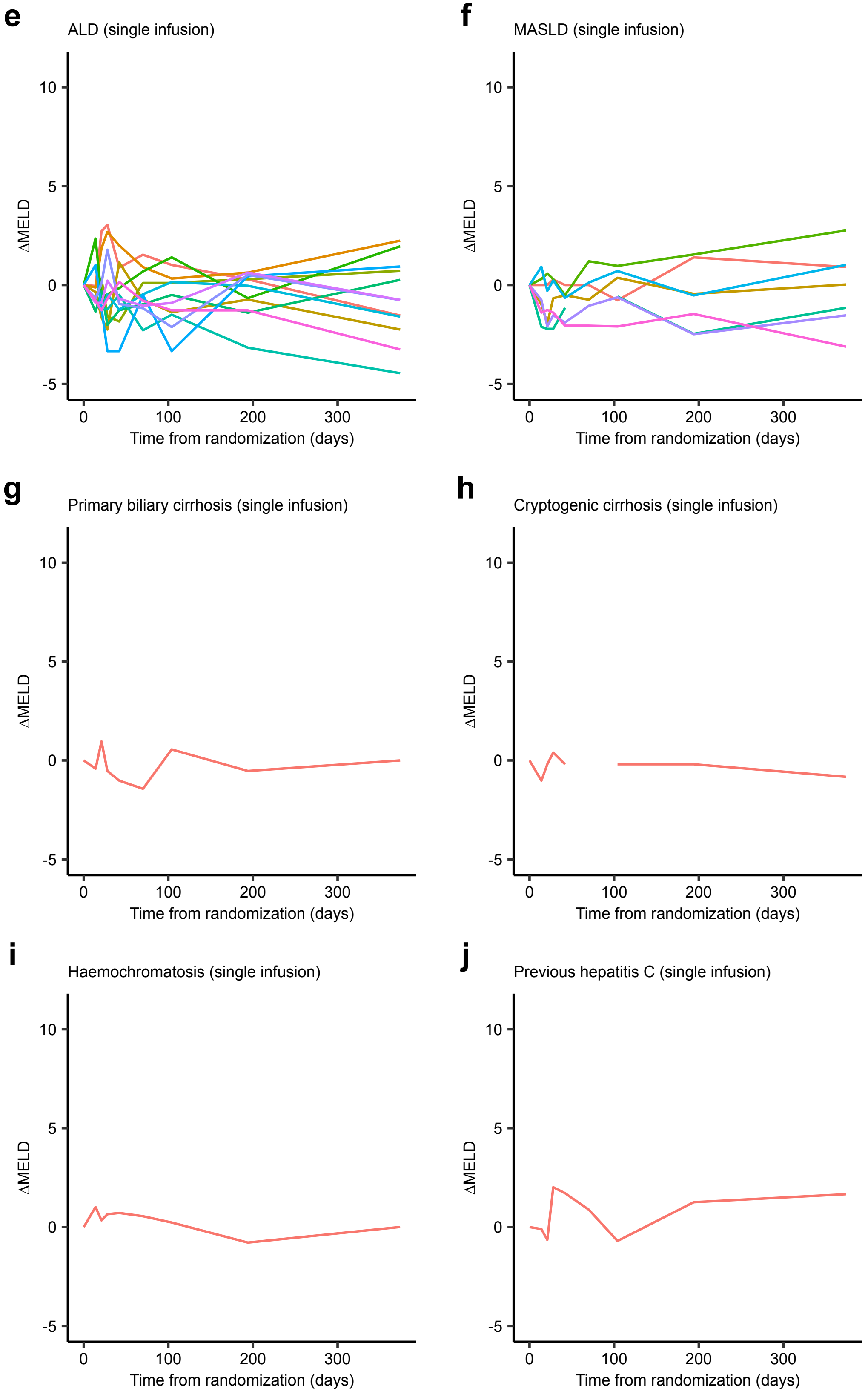
e
ALD (single infusion)
f
MASLD (single infusion)
g
Primary biliary cirrhosis (single infusion)
h
Cryptogenic cirrhosis (single infusion)
i
Haemochromatosis (single infusion)
j
Previous hepatitis C (single infusion)
ΔMELD
10
5
0
-5
0
100
200
300
Time from randomization (days)

k

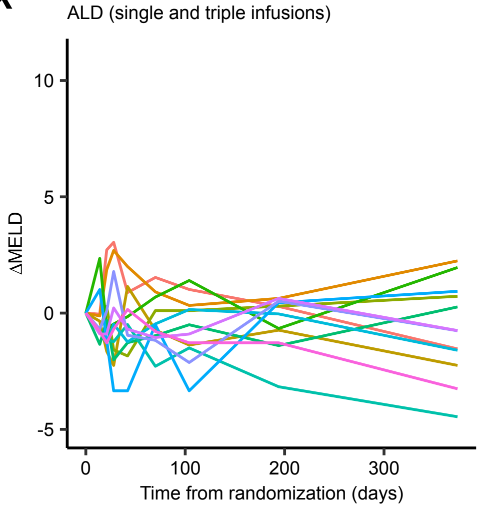

ALD (single and triple infusions)
ΔMELD
10
5
0
-5
0
100
200
300
Time from randomization (days)

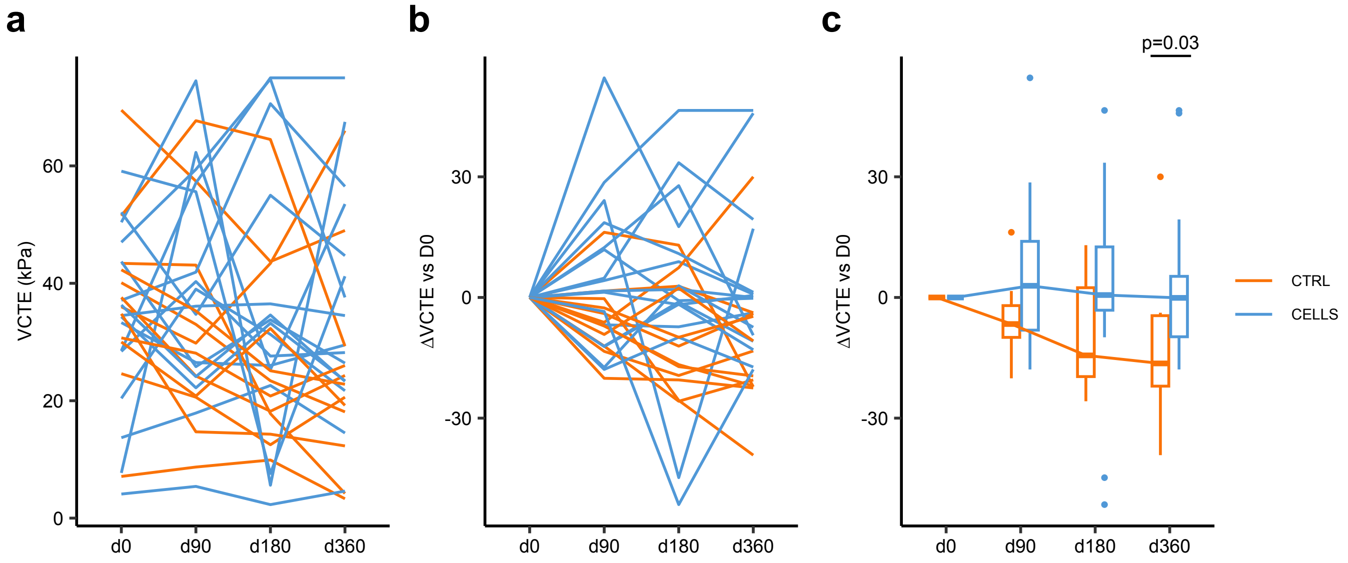
a
b
c
VCTE (kPa)
0
20
40
60
d0
d90
d180
d360
ΔVCTE vs D0
-30
0
30
p=0.03
CTRL
CELLS

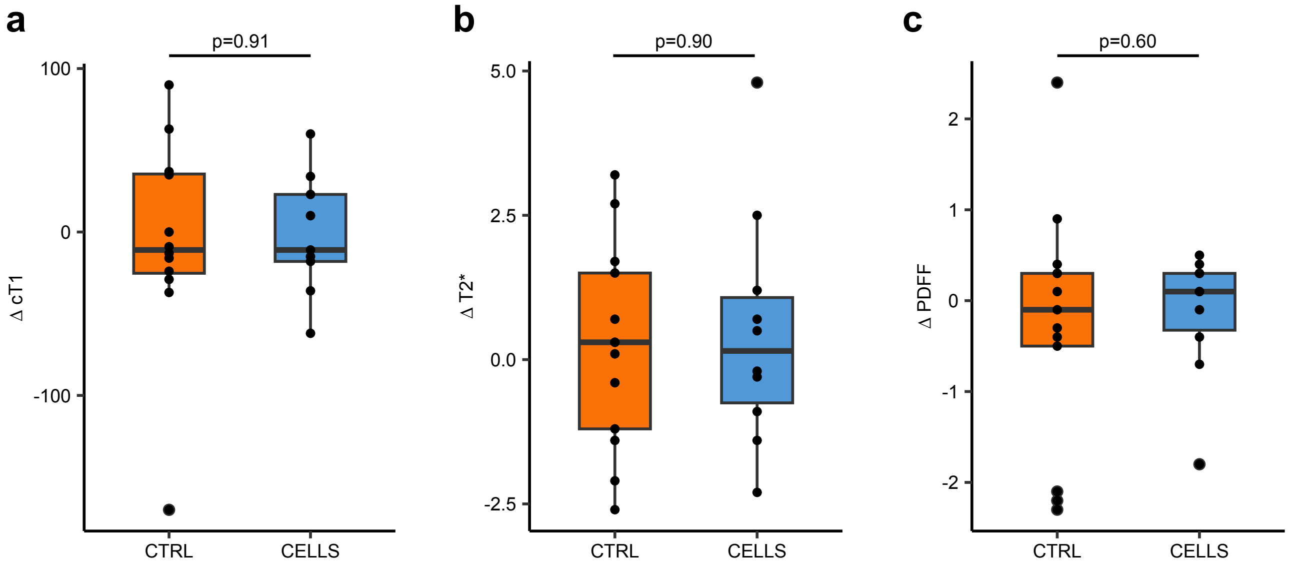

a
p=0.91
100
0
-100
Δ cT1
CTRL
CELLS
b
p=0.90
5.0
2.5
0.0
-2.5
Δ T2*
CTRL
CELLS
c
p=0.60
2
1
0
-1
-2
Δ PDFF
CTRL
CELLS

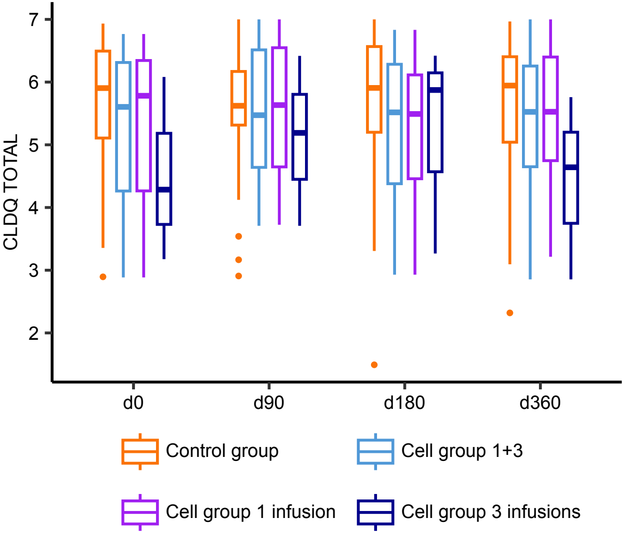
CLDQ TOTAL
7
6
5
4
3
2
d0
d90
d180
d360
Control group
Cell group 1+3
Cell group 1 infusion
Cell group 3 infusions

	Randomized treatment	N	N missing	Mean	SD	Min.	Lower quartile	Median	Upper Quartile	Max
**cT1 90 day**	Cell group 1 infusion	10	14	970.6	76.69	839	911	981.5	1000	1107
	Control group	12	12	936.5	120.7	777	801	951.5	1022	1115
**cT1 baseline**	Cell group 1 infusion	9	15	971.4	83.16	816	931	966	1018	1105
	Control group	13	11	936.9	134.5	793	833	888	1022	1265
**ΔcT1 (base to 90 day)**	Cell group 1 infusion	9	15	-1.67	37.37	-62	-18	-11	23	60
	Control group	12	12	-6.08	64.96	-170	-26.5	-11	36	90

	Randomized treatment	N	N missing	Mean	SD	Min	Lower quartile	Median	Upper Quartile	Max
ROI T2* 90 day	Cell group 1 infusion	9	15	23.41	3.74	18.8	21.1	21.7	25.4	30.8
ROI T2* 90 day	Control group	12	12	19.36	5.57	6.4	16.1	21.25	23.55	25.1
ROI T2* baseline	Cell group 1 infusion	10	14	22.81	3.61	18.3	20.9	22.6	24.2	31.1
ROI T2* baseline	Control group	12	12	20.13	4.92	7.6	18.4	22	23.5	24.8
Δ ROI T2* (base to 90 day)	Cell group 1 infusion	9	15	0.61	2.11	-2.3	-0.3	0.5	1.2	4.8
Δ ROI T2* (base to 90 day)	Control group	11	13	0.11	1.89	-2.6	-1.4	0.3	1.7	3.2

	Randomized treatment	N	N missing	Mean	SD	Min	Lower quartile	Median	Upper Quartile	Max
PDFF 90 day	Cell group 1 infusion	10	14	3.76	2.6	1.7	2.1	2.75	4.1	9.8
PDFF 90 day	Control group	10	14	4.16	2.88	1.6	2.3	3.05	5.8	9.5
PDFF baseline	Cell group 1 infusion	9	15	4.1	3.11	1.4	2.3	2.7	4.8	9.9
PDFF baseline	Control group	11	13	4.92	3.62	1.6	1.9	3.7	8.1	11.6
∆PDFF (base to 90 day)	Cell group 1 infusion	9	15	-0.16	0.73	-1.8	-0.4	0.1	0.3	0.5
∆PDFF (base to 90 day)	Control group	8	16	-0.98	1.03	-2.3	-2.15	-0.45	-0.2	0.1

Supplementary Fig. 1. Individual participant MELD score changes from baseline to day 360 (∆MELD).

a-d, ∆MELD score from baseline, split by dominant etiology; control group.

e-j, ∆MELD score from baseline, split by dominant etiology; single infusion group.

k, ∆MELD score from baseline, split by dominant etiology – ALD; single and triple infusion groups. There were no additional dominant etiologies for the triple infusion group.

Supplementary Fig. 2. Vibration-controlled transient elastography of MATCH01 trial participants. a, Plot of vibration-controlled transient elastography (VCTE) measurements from the Fibroscan^{TM} platform for each participant where available, measured at day 0, 90, 180 and 360 time points. Lines are coloured according to allocated treatment group. **b,** Plot of ∆VCTE for each patient, compared to day 0, at each time point. **c,** Boxplots of ∆VCTE at each time point, split by treatment group. Ctrl group: n=12 participants. Cell group: n=16 participants (this group includes one triple infusion participant). Lines connect group medians. Repeated ANOVA, two-sided, with pairwise two-sided post-hoc tests. Boxplots are defined as first to third quartile (Q1, Q3), with centre line representing the median. Whiskers extend to the lowest/highest values no further than 1.5*IQR from Q1/Q3, as appropriate.

Supplementary Fig. 3. Multiparametric magnetic resonance imaging of MATCH01 trial participants. Boxplots of delta **(a)** corrected T1 (∆cT1), **(b)** T2* (∆T2*) and **(c)** proton density fat fraction (∆PDFF) participant measurements from Liver*MultiScan*® platform where available, split by treatment group. Ctrl group: n=13 participants (except ∆cT1 n=12). Cells group: n=10 participants (except ∆cT1 n=9); cells group contains only single-infusion participants for all measurements. Wilcoxon rank sum exact test, two-sided. Boxplots are defined as first to third quartile (Q1, Q3), with centre line representing the median. Whiskers extend to the lowest/highest values no further than 1.5*IQR from Q1/Q3, as appropriate.

Supplementary Fig. 4. Total Chronic Liver Disease Questionnaire (CLDQ) score of MATCH01 participants. Total CLDQ score, where available, in participants who received standard care (control; n=21), cell group single and triple infusions (n=26), Cell group single infusion (n=23) and cell group triple infusions (n=3) from day 0 to day 360. Boxplots are defined as first to third quartile (Q1, Q3), with centre line representing the median. Whiskers extend to the lowest/highest values no further than 1.5*IQR from Q1/Q3, as appropriate.

Supplementary Table 1. Liver cT1 measurements from Liver*MultiScan*® platform. Descriptive statistics for liver cT1, at baseline, day 90 and delta.

Supplementary Table 2. Liver ROI T2* measurements from Liver*MultiScan*® platform. Descriptive statistics for liver ROI T2*, at baseline, day 90 and delta.

Supplementary Table 3. Liver PDFF measurements from Liver*MultiScan*® platform. Descriptive statistics for liver PDFF, at baseline, day 90 and delta.
