# Supplementary material for: Autologous macrophage therapy for liver cirrhosis: a phase 2 open-label randomized controlled trial
Source: Nat Med. 2025 Jan 10;31(3):979–87. doi: 10.1038/s41591-024-03406-8 (PMC11922741; doi:10.1038/s41591-024-03406-8)
Supplement: Supplementary file 2 — Reporting Summary [file 41591_2024_3406_MOESM2_ESM.pdf]

Corresponding author(s): Stuart J Forbes

Last updated by author(s): Nov 4, 2024

Reporting Summary

Nature Portfolio wishes to improve the reproducibility of the work that we publish. This form provides structure for consistency and transparency in reporting. For further information on Nature Portfolio policies, see our Editorial Policies and the Editorial Policy Checklist.

Statistics

For all statistical analyses, confirm that the following items are present in the figure legend, table legend, main text, or Methods section.

n/a	Confirmed	
☐	☒	The exact sample size (*n*) for each experimental group/condition, given as a discrete number and unit of measurement
☐	☒	A statement on whether measurements were taken from distinct samples or whether the same sample was measured repeatedly
☐	☒	The statistical test(s) used AND whether they are one- or two-sided<br>*Only common tests should be described solely by name; describe more complex techniques in the Methods section.*
☐	☒	A description of all covariates tested
☐	☒	A description of any assumptions or corrections, such as tests of normality and adjustment for multiple comparisons
☐	☒	A full description of the statistical parameters including central tendency (e.g. means) or other basic estimates (e.g. regression coefficient) AND variation (e.g. standard deviation) or associated estimates of uncertainty (e.g. confidence intervals)
☐	☒	For null hypothesis testing, the test statistic (e.g. *F*, *t*, *r*) with confidence intervals, effect sizes, degrees of freedom and *P* value noted<br>*Give P values as exact values whenever suitable.*
☒	☐	For Bayesian analysis, information on the choice of priors and Markov chain Monte Carlo settings
☒	☐	For hierarchical and complex designs, identification of the appropriate level for tests and full reporting of outcomes
☐	☒	Estimates of effect sizes (e.g. Cohen's *d*, Pearson's *r*), indicating how they were calculated

Our web collection on statistics for biologists contains articles on many of the points above.

Software and code

Policy information about availability of computer code

Data collection	N/A
Data analysis	N/A

For manuscripts utilizing custom algorithms or software that are central to the research but not yet described in published literature, software must be made available to editors and reviewers. We strongly encourage code deposition in a community repository (e.g. GitHub). See the Nature Portfolio guidelines for submitting code & software for further information.

Data

Policy information about availability of data

All manuscripts must include a data availability statement. This statement should provide the following information, where applicable:

- Accession codes, unique identifiers, or web links for publicly available datasets
- A description of any restrictions on data availability
- For clinical datasets or third party data, please ensure that the statement adheres to our policy

We have included a succinct data availability statement within the manuscript

Human research participants

Policy information about studies involving human research participants and Sex and Gender in Research.

Reporting on sex and gender	"Gender" is recorded as "male" or "female" for purposes of demographics and is consistent with "sex" at birth.
Population characteristics	No specific relevant ethinic considerations of note.
Recruitment	Patients were recruited from 3 liver centres across Scotland. Royal Infirmary of Edinburgh, Glasgow Royaly Infirmary and Ninewells Hospital and Medical School. Patients were screened from cohorts of patients in the representative centres, and approached by research physician or nurse if they met the minimal inclusion criteria.<br>As such, there was no prescriptive biases at time of invitation, or screening, whilst during randomisation there was minimisation algorithm allocation based on etiological variant. Other than the risk of self-selection bias inherent in enrolling in a clinical trial all other variables were adequately controlled for in so far as possible within the trial design.
Ethics oversight	The trial was approved by Scotland A Research Ethics Committee (Reference: 15/SS/0121), NHS Lothian Research and Development.

Note that full information on the approval of the study protocol must also be provided in the manuscript.

Field-specific reporting

Please select the one below that is the best fit for your research. If you are not sure, read the appropriate sections before making your selection.

☒ Life sciences ☐ Behavioural & social sciences ☐ Ecological, evolutionary & environmental sciences

For a reference copy of the document with all sections, see nature.com/documents/nr-reporting-summary-flat.pdf

Life sciences study design

All studies must disclose on these points even when the disclosure is negative.

Sample size	Predicted sample size was 56 persons with anticipated drop-out rate of 10.
Data exclusions	Only single individual drop out.
Replication	N/A
Randomization	Dedicated software (RedCap), based on minimisation algorithm using etiologies (ALD, NAFLD, Other) in 1:1:1 ratio
Blinding	Open Label

Reporting for specific materials, systems and methods

We require information from authors about some types of materials, experimental systems and methods used in many studies. Here, indicate whether each material, system or method listed is relevant to your study. If you are not sure if a list item applies to your research, read the appropriate section before selecting a response.

Materials & experimental systems

n/a	Involved in the study	
☒	☐	Antibodies
☒	☐	Eukaryotic cell lines
☒	☐	Palaeontology and archaeology
☒	☐	Animals and other organisms
☐	☒	Clinical data
☒	☐	Dual use research of concern

Methods

n/a	Involved in the study	
☒	☐	ChIP-seq
☒	☐	Flow cytometry
☒	☐	MRI-based neuroimaging

Clinical data

Policy information about clinical studies

All manuscripts should comply with the ICMJE guidelines for publication of clinical research and a completed CONSORT checklist must be included with all submissions.

Clinical trial registration	ISRCTN10368050 and EudraCT Reference: 2015-000963-15
Study protocol	Previously published, PMID 34750149
Data collection	Standard template with bespoke secure data management system (REDCAP)
Outcomes	As described within the statistical analysis plan, the primary endpoint was the ΔMELD assessed using a two-sample t-test (with Welch correction for unequal variance).<br>The relevant secondary endpoints included assessment of the changes from D0 to D90 for Enhanced Liver Function (ELF) test, PRO-C3, C3M, UKELD score, vibration controlled transient elastography (VCTE), multiparametric MRI scanning and quality of life assessment using CLDQ-9. We assessed exploratory changes in cytokine and chemokine expression.
